# Supplementary material for: Novel Driver Strength Index highlights important cancer genes in TCGA PanCanAtlas patients
Source: PeerJ. 2022 Aug 11;10:e13860. doi: 10.7717/peerj.13860 (PMC9375969; doi:10.7717/peerj.13860)
Supplement: Supplemental Information 2 [file peerj-10-13860-s002.zip › COHORTS/genes plots/cohorts/2021_8_16_14_9_distribution_cohorts_males_KICH.pdf]

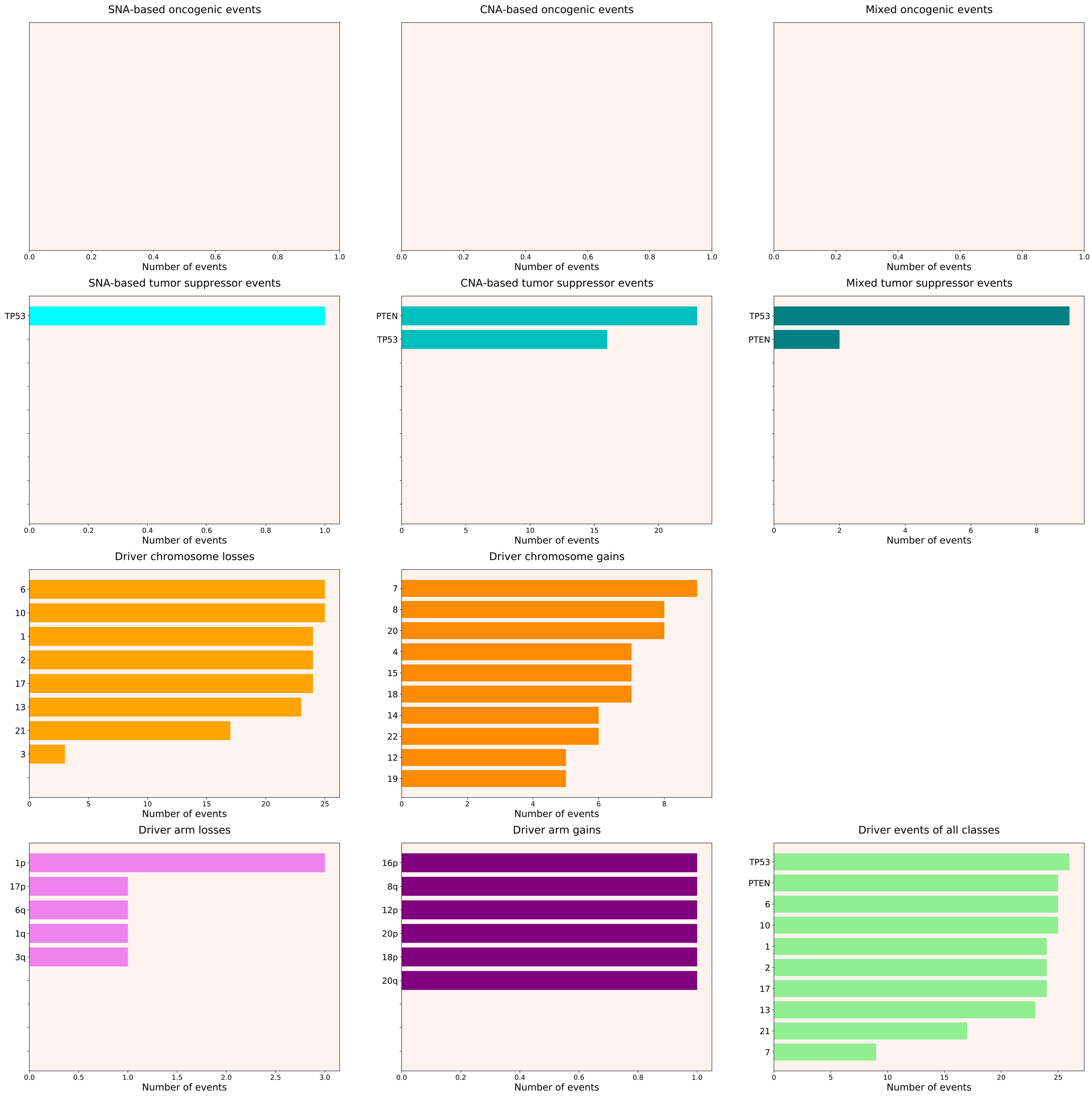

SNA-based oncogenic events
CNA-based oncogenic events
Mixed oncogenic events
SNA-based tumor suppressor events
TP53
CNA-based tumor suppressor events
PTEN
TP53
Mixed tumor suppressor events
TP53
PTEN
Driver chromosome losses
6
10
1
2
17
13
21
3
Driver chromosome gains
7
8
20
4
15
18
14
22
12
19
Driver arm losses
1p
17p
6q
1q
3q
Driver arm gains
16p
8q
12p
20p
18p
20q
Driver events of all classes
TP53
PTEN
6
10
1
2
17
13
21
7
Number of events
